# Genetic associations and shared environmental effects on the skin microbiome of Korean twins

**DOI:** 10.1186/s12864-015-2131-y

**Published:** 2015-11-23

**Authors:** Jiyeon Si, Sunghee Lee, Jin Mo Park, Joohon Sung, GwangPyo Ko

**Affiliations:** Department of Environmental Health, School of Public Health, Seoul National University, 1 Gwanak-ro, Gwanak-gu, Seoul, 151-742 Korea; Cutaneous Biology Research Center, Massachusetts General Hospital and Harvard Medical School, Charlestown, MA 02129 USA; Department of Epidemiology, School of Public Health, Seoul National University, 1 Gwanak-ro, Gwanak-gu, Seoul, 151-742 Korea

**Keywords:** Skin microbiota, Heritability, Twin study, Host genetics, Common environment effect

## Abstract

**Background:**

The skin is the outermost layer of the human body and one of the key sites for host-microbe interactions. Both environmental and host genetic factors influence microbial communities in distinct anatomical niches, but little is known about their interplay in shaping the skin microbiome. Here, we investigate the heritable components of the skin microbiome and their association with host genetic factors.

**Results:**

Based on our analysis of the microbiota from 45 individuals including monozygotic and dizygotic twins aged 26–55 years and their mothers, we found that skin microbial diversity was significantly influenced by age and skin pigmentation. Heritability analysis revealed genetic and shared environmental impacts on the skin microbiome. Furthermore, we observed a strong association between the abundance of *Corynebacterium jeikeium* and single nucleotide polymorphisms (SNPs) in the host FLG gene related to epidermal barrier function.

**Conclusion:**

This study reveals an intimate association of the human skin microbiome and host genes, and increases our understanding of the role of human genetic factors in establishing a microbial ecosystem on the body surface.

**Electronic supplementary material:**

The online version of this article (doi:10.1186/s12864-015-2131-y) contains supplementary material, which is available to authorized users.

## Background

The skin provides a protective barrier against invading microbial pathogens, while also serving as a habitat for a plethora of commensal bacteria. The composition of skin microbiota varies among individuals, but a core set of microbes can be found in all subjects for each anatomical location [1–3]. Recent advances in high-throughput sequencing technologies have enabled rapid analysis of microbial communities indigenous to specific niches in the human body [4]. There is significant inter-individual variation in the skin microbiota, which is attributable to genetic differences and environmental factors. Colonization of the host body by bacteria occurs immediately after birth, with more complex microbial communities developing in distinct anatomical niches afterwards [5]. Succession of the microbiota within individual hosts is also driven by host-intrinsic factors, such as age, gender, genotype and health status, as well as environmental and lifestyle factors, such as climate, light exposure, and detergent use [6, 7]. Previous studies have quantitatively explored host genetic effects on the gut microbiome composition using animal models and humans [8–11]. These studies revealed a significant influence of host genetic factors on the microbiota. However, the impact of host gene expression on skin microbial diversity in human subjects has not been fully investigated.

The majority of complex human traits are governed by multiple gene interactions [12]. These sophisticated interactions differ among individuals based on environmental factors. Studies on twins are therefore a useful method for estimating the genetic and environmental effects that a given factor has on human health [13]. Twin heritability and linkage studies segregate the genetic and environmental effects based on the assumption that monozygotic twins are genetically identical, and so their phenotypic differences are of environmental origin. Additionally, the shared environmental effects can be determined from monozygotic (MZ) and dizygotic (DZ) twins, as well as parent-offspring pairs [13]. Our knowledge of individual differences in the skin microbiota and genetic markers associated with its composition remains limited. In this study, we analyzed the skin microbiota of 16 MZ and 8 DZ twins between 26 and 55 years of age and their mothers (*n* = 16) to identify the heritable components of the skin microbiome and their association with host genetic factors. To this end, we focused our analysis on host genes related to key dermatological conditions, including sebum production, skin humidity, pigmentation, epidermal barrier function, and hair follicle development. Using the microbial composition as quantitative traits, we identified one human single nucleotide polymorphisms (SNPs) strongly associated with the abundance of *Corynebacterium jeikeium* on the skin.

## Results and discussion

### Study population and composition of the human skin microbiome

**Fig. 1:**
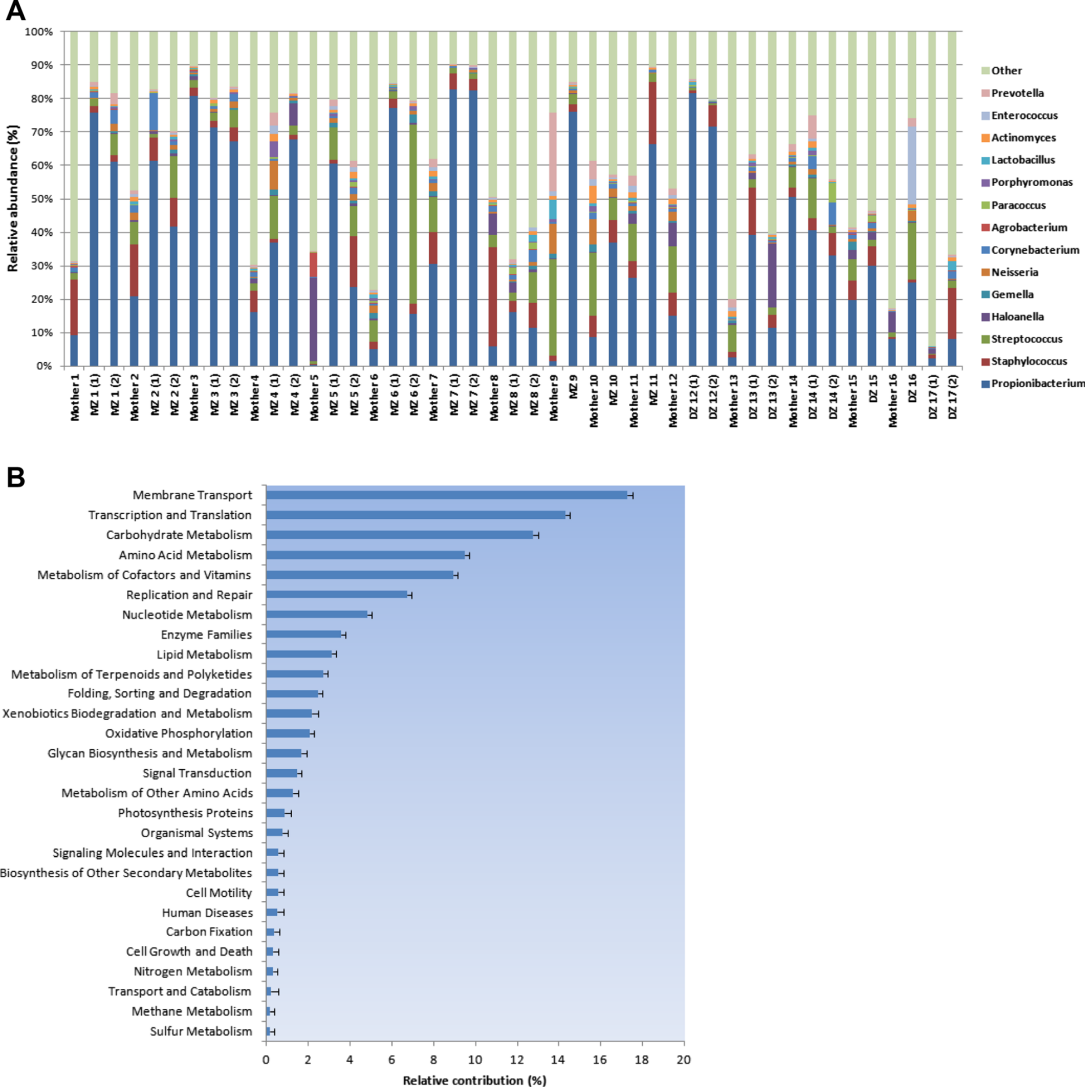
Diversity of the skin microbiota and functional traits of the microbial communities. **a** Taxonomic classification of the skin microbiome at the genus level. Relative bacterial abundance for each individual is shown. A familial relationship is indicated among those individuals represented by the same number. Twin pairs are presented in the parenthesis. **b** Skin metagenomes predicted using PICRUSt (Methods). Relative contribution of each functional pathway is determined for the collective microbiota of all subjects. Error bars, SEM

The study population included Korean subjects (*n* = 45) with eight MZ and four DZ twin pairs, five singletons, and 16 of their mothers (Table 1). Skin traits including skin humidity and pigmentation were measured in 32 subjects. Subjects were categorized as having sufficiently moisturized versus dry skin, and mid- versus light-tone skin color based on threshold values in an arbitrary unit for skin humidity (45 AU) and pigmentation (150 AU), respectively. A total of 860,547 sequencing reads from the V2-V3 regions of the human 16S rRNA genes were generated from 45 skin swab samples, for an average of 19,123 reads per sample. We chose the flexor surface of the right upper arm to minimize variations due to differences in the use of cosmetics, surface temperature, acidity, humidity, handedness, and other external factors. The skin microbiota was first assessed at the phylum level; Actinobacteria (50.0 %), Firmicutes (22.0 %), Proteobacteria (16.1 %), and Bacteroidetes (6.0 %) were the dominant phyla among the samples collected. The composition of the skin microbiome in our study was consistent with previous reports [7]. Further classification of the skin microbiota at the genus level revealed high inter-individual variation with *Propionibacterium* (37.11 %), *Staphylococcus* (6.79 %), and *Streptococcus* (6.95 %) prevailing on the skin (Fig. 1a). At the species level, *P. acnes* was the most abundant inhabitant, accounting for 23.62 % of the skin microbiota. Occupancy by the remaining species accounted for less than 1 % each.

**Table. 1:** Summary of the study population (*N* = 45)

	Mother	MZ twin	DZ twin	Singleton^a^	Sum
No.	16	16	8	5	45
Age, mean (SD), y	62.1 (8.5)	31.0 (3.6)	36.8 (5.1)	41.2 (8.4)	
Sex					
Female, no.	16	10	4	3	33
Male, no.	-	6	4	2	12
Humidity (SD), AU^bc^	30.1 (20.6)	36.3 (29.3)	19.0 (21.3)	28.7 (17.3)	
Pigmentation (SD), AU^bd^	124.3 (83.4)	97.9 (72.3)	65.6 (73.1)	111.6 (64.8)	

^a^Three MZ and two DZ twins were analyzed as singletons, since one of their associated twins was excluded due to antibiotic use

^b^Dermatologic phenotypes were measured from 12 mothers, 15 MZ twins, and 5 DZ twins

^c^The level of skin humidity is expressed using arbitrary units (AU) as given by the device (Corneometer®CM825). Corneometry values greater than 45 AU indicate sufficiently moisturized skin, while values less than 45 AU indicate dry skin

^d^A melanin index of 0–150 AU indicates light skin tone, and values of 150–250 AU indicate mid-tone skin pigmentation

To explore the skin microbe-host interaction further, we used PICRUSt [14], an algorithm that detects the functional capabilities of a community by comparing its metagenome with reference genomes using microbial communities identified from all subjects. PICRUSt classified 200 functional pathways from the 16S rRNA sequences of human skin microbiota.

Functional traits determined based on 16S rRNA genes showed categorical similarity with a recent skin metagenomic study (Fig. 1b), with a large proportion of the functions associated with carbohydrate, amino acid, vitamin, and nucleotide metabolism [15]. Discrepancies in the relative contribution of each pathway between the studies could be due to use of different sampling sites and analytical methods. The previous metagenomic study reflects the microbial functions from two male subjects at mid-twenties with samples from five different body parts. Additionally, 16S rRNA based predictions of the functional traits by PICRUSt could possibly cause the discrepancies. Detailed categories of the functional traits and Nearest Sequenced Taxon Index (NTSI) values are provided in Additional file 1: Table S1 and Additional file 1: Table S2, respectively.

### Diversity of the human skin microbiome

**Fig. 2:**
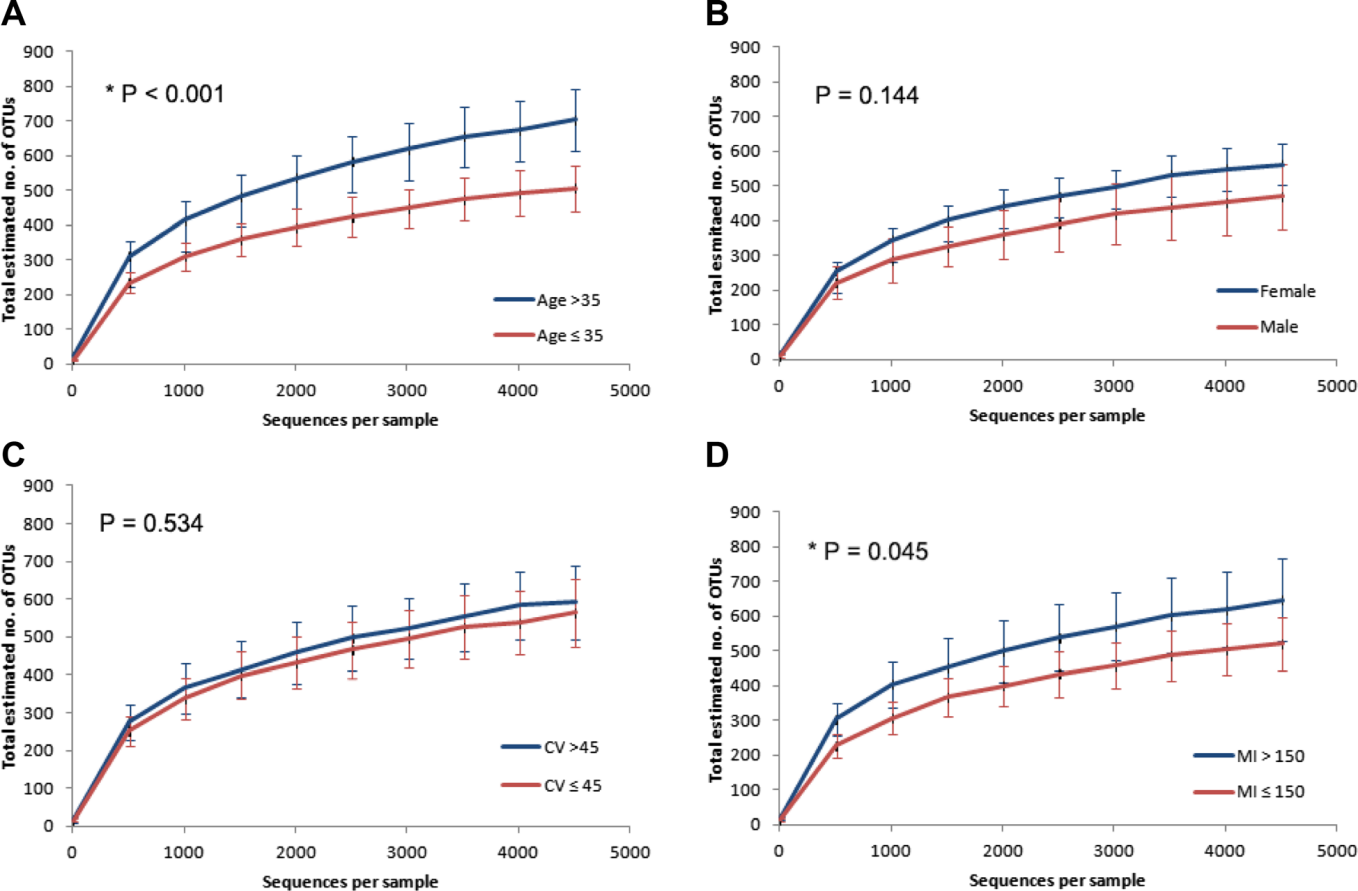
Differences in skin microbial diversity analyzed using rarefaction curves **a** Age. **b** Gender. **c** Skin humidity (*CV, Corneomerty Value*). **d** Pigmentation (*MI, Melanin Index*). Error bars indicate 95 % confidence intervals. * Significance is based on the Wilcoxon rank-sum test

Microbial diversity “within” and “between” subject groups is denoted by alpha and beta diversity, respectively. In an assessment of species richness, the rarefaction curves, reflective of alpha diversity, showed significantly higher levels of bacterial diversity at ages greater than 35 years (Wilcoxon rank-sum test, *W* = 109, *P* < 0.001; Fig. 2). The median value was used as the cutoff age at 35 to evenly disperse individuals into two groups. Previous studies have shown that age is a primary contributor to increased microbial diversity in the skin. A study comparing the skin microbiota between children and adolescents revealed microbial shifts with the onset and progression of sexual maturation [16]. The amount of sebaceous wax ester is known to be closely associated with age, increasing between the ages of 15–35 years and decreasing afterwards [17]. An increase in skin lipids is thought to create an acidic environment, reducing the numbers of acid-susceptible bacteria—such as Staphylococcal and Streptococcal species—on adolescent skin. Disparities in sweat, sebum, and hormone production, as well as lifestyle choices such as the use of cosmetics, may underlie gender differences in skin microbiota [2, 3, 15]. However, gender effects were not present on the inner wrist (Wilcoxon rank-sum test, *W* = 147, *P* = 0.144) when mothers were excluded to avoid confounding by age. The gender effects on bacterial diversity seem to depend on the skin site; greater diversity of bacteria in females was evident on hands [2] but not at the base of the neck [18]. Additionally, we could not observe the significant effect of skin humidity (Wilcoxon rank-sum test, *W* = 126, *P* = 0.534) on the skin. This is likely due to the relatively low variation in humidity in the skin of the upper arm where the microbiota was sampled. Interestingly, skin pigmentation significantly influenced skin bacteria, increasing the diversity in individuals with an intermediate skin tone (Wilcoxon rank-sum test, *W* = 176, *P* = 0.045; Additional file 1: Figure S1). While there have been reports on the effects of bacterial species on melanogenesis [19–21], the exact nature of the relationship between the skin microbiota and pigmentation remains to be determined. Our data revealed only a weak association with skin humidity (Pearson correlation, *r* = 0.393, *P* = 0.029; Additional file 1: Table S3). To compare the beta diversity profiles across different status of age, gender, skin humidity, and pigmentation, we performed a principal coordinate analysis (PCoA) using weighted and unweighted UniFrac metrics (Additional file 1: Figure S2), wherein the distance between microbial communities was calculated based on phylogenetic information. The results were not indicative of complete separation of microbiotas based on the host phenotypes (ANOSIM, *P* > 0.05 for all host traits) indicating that the groups divided by age, gender, skin humidity, and pigmentation did not include significantly different microbial compositions.

### Heritability of the skin microbiota

**Fig. 3:**
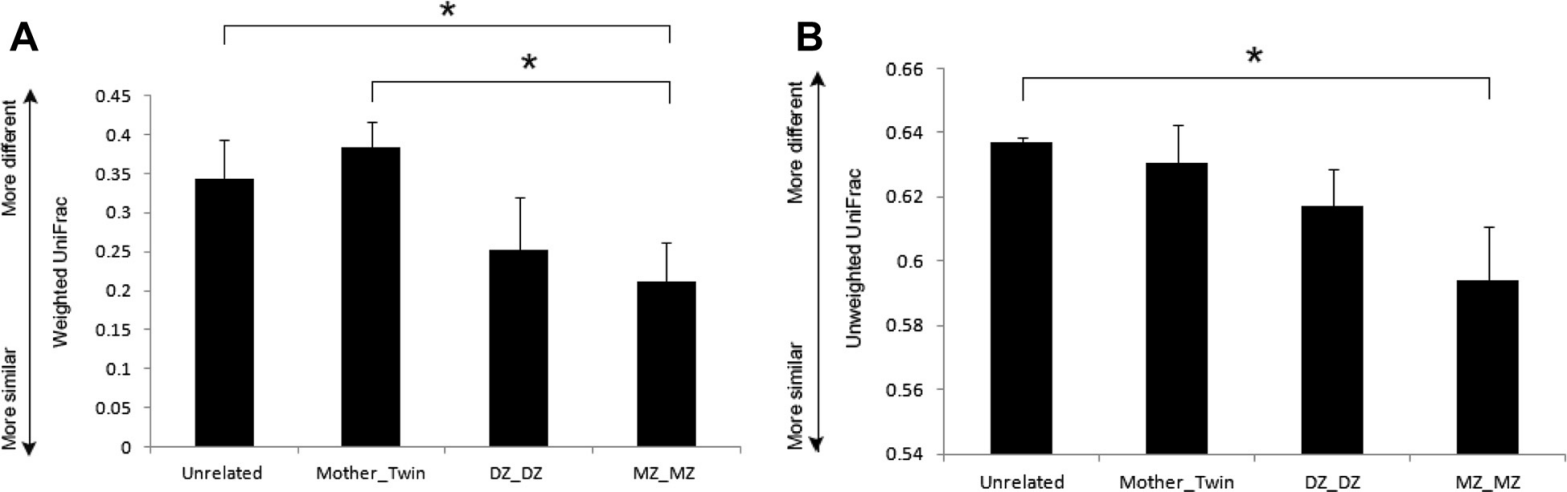
Comparison of the skin microbiota of twins and their families in terms of heritability and environmental effects. **a** Weighted and **b** unweighted UniFrac distance between twin pairs (4 UniFrac distance values for DZ pairs and 8 for MZ pairs), twins and mothers (25 UniFrac distance values), and unrelated individuals (909 UniFrac distance values). The error bars represent the standard error. *,*P*< 0.05 based on Wilcoxon rank sum test

Comparison of the skin microbiota between twins and their mothers was performed using weighted and unweighted UniFrac distances (Fig. 3a and b). With both metrics, MZ twins showed the highest similarity, followed by DZ twins and mother-twins. The dissimilarity of unrelated subject was the highest under the unweighted UniFrac metrics whereas it was the second highest next to mother-twins under the weighted UniFrac distance. The similarity of the skin microbiota of MZ twins was comparable to that of DZ twins, but significantly higher than those in unrelated subjects and their mothers (*P* < 0.05). Our subsequent analysis of the skin microbiota focused on heritability and the additional effects stemming from common environments (Table 2). Heritability ranged from 40.9–56.4 % with the abundance of *Roseomonas* in the skin showed the highest degree of heritability. Although the role is not well-defined, a recent study comparing skin microbes on the face and other body parts found an increased abundance of *Roseomonas* on the scapula and inner elbow [22]. Two of the taxonomic branches described in this study were found to possess a heritable component: Corynebacteriaceae/ *Corynebacterium* and Brevibacteriaceae/ *Brevibacterium*/ *Brevibacterium aureum. Corynebacterium* is a major constituent of the normal skin flora along with *Staphylococcus*, *Propionium*, *Streptococcus,* and *Pseudomonas* [23]*. C. jeikeium*, the only species belonging to genus *Corynebacterium* described in this study, is commonly found on skin epithelium, and it has been shown to cause nosocomial infections in immunocompromised patients [24, 25]. *Brevibacterium* sp. is a Gram-negative bacterium capable of degrading cyfluthrin, an organic compound with xenobiotic characteristics and toxicity to the reproductive, neural, and respiratory systems in humans [26]. The range of shared-environmental effects on the skin microbiome was 56–72.9 %, with Leuconostocaceae/ *Weissella/ Weissella cibaria* (70.1 %; *P* = 0.021) and Propionibacteriaceae/ *Propionibacterium*/ *Propionibacterium acnes* (57.7 %; *P* = 0.027) significantly influenced by the common environment from family to species. *W. cibaria* is a lactic acid bacterium belonging to the family of Leuconostocaceae [27]. Lactic acid bacteria require specific nutrients, such as carbohydrates, amino acids, vitamins, purines, and pyrimidines for growth [28]. Despite these requirements, the bacteria have been isolated from a variety of environments, including the oral cavity, intestinal tract, and fermented foods [28, 29]. This adaptability likely accounts for the presence of the bacteria in two seemingly incongruous locations. *P. acnes*, the most dominant and well-known skin commensal bacterium [30], is heavily reliant on the metabolism of fatty acids found in sebum secretions. Soap, detergents, astringents, and other factors that alter the amount of sebum may account for the environmental effects on *P. acnes* colonization [31]. Individual-specific effects ranged from 29.9–59.1 %.

**Table. 2:** Heritability and household effects of the skin microbiota after adjustment for age and gender

	Genetic Effect (95 % CI)	*P*-value	Common Environment Effect (95 % CI)	*P*-value	Individual Effect (95 % CI)
f__Brevibacteriaceae	0.409 (0.191–0.627)	0.047			0.59 (0.372–0.808)
f__Bacillaceae	0.465 (0.213–0.717)	0.048			0.535 (0.283–0.787)
f__Corynebacteriaceae	0.468 (0.273–0.663)	0.019			0.532 (0.337–0.727)
g__Corynebacterium	0.468 (0.273–0.663)	0.019			0.532 (0.337–0.727)
g__Peptoniphilus	0.426 (0.216–0.636)	0.034			0.573 (0.363–0.783)
g__Brevibacterium	0.409 (0.191–0.627)	0.047			0.59 (0.372–0.808)
g__Roseomonas	0.564 (0.333–0.795)	0.033			0.436 (0.205–0.667)
*s__Brevibacterium aureum*	0.409 (0.191–0.627)	0.047			0.59 (0.372–0.808)
c__Sphingobacteria			0.659 (0.367–0.951)	0.021	0.246 (0.1–0.392)
f__Hyphomicrobiaceae			0.611 (0.436–0.786)	0.025	0.389 (0.214–0.564)
f__Leuconostocaceae			0.729 (0.594–0.864)	0.011	0.271 (0.136–0.406)
f__Microbacteriaceae			0.626 (0.437–0.815)	0.032	0.373 (0.184–0.562)
f__Propionibacteriaceae			0.563 (0.366–0.760)	0.028	0.437 (0.240–0.634)
f__Rhodospirillaceae			0.563 (0.366–0.762)	0.031	0.436 (0.237–0.635)
f__Sphingobacteriaceae			0.560 (0.366–0.756)	0.048	0.44 (0.244–0.636)
g__Propionibacterium			0.563 (0.366–0.760)	0.028	0.437 (0.240–0.634)
g__Weissella			0.701 (0.546–0.856)	0.021	0.299 (0.144–0.454)
*s__Propionibacterium acnes*			0.577 (0.384–0.770)	0.027	0.422 (0.229–0.615)
*s__Weissella cibaria*			0.701 (0.546–0.856)	0.021	0.299 (0.144–0.454)

Each taxonomic level is indicated by f, g, and s for family, genus, and species, respectively

### Genetic association of skin microbiotas

**Fig. 4:**
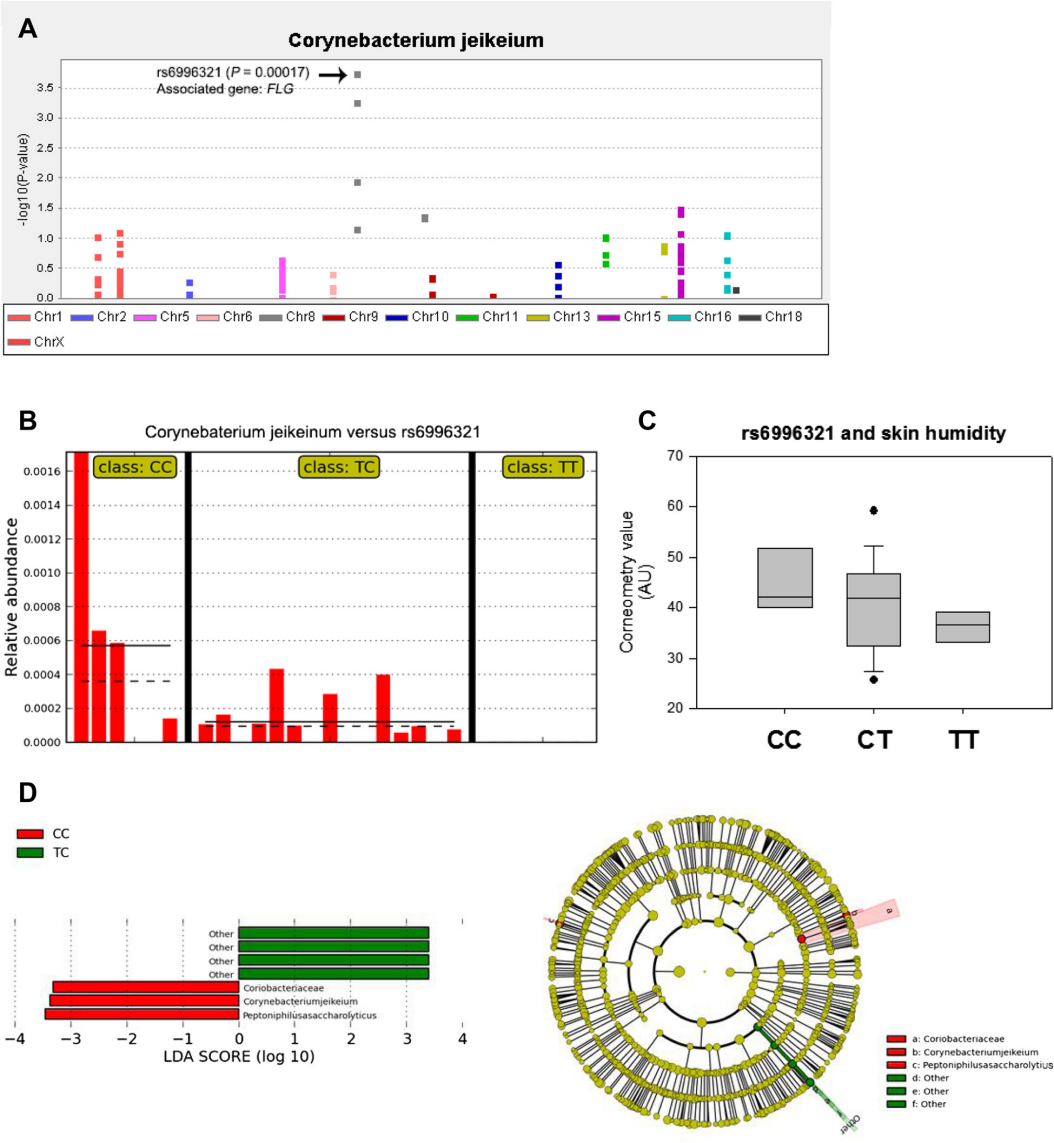
Effect of the host genotype on skin traits. **a** Manhattan plot summarizing the results of an association analysis of 275 candidate SNPs with *Corynebacterium jeikeium*. Each dot represents the candidate SNP plotted across the genome. **b** Relative abundance of *C. jeikeium* with respect to rs6996321. The means and standard errors are indicated by solid and dashed bars, respectively. **c** Level of skin humidity by genotype at rs6996321. Boxes represent the 25^th^ percentile, median, and 75^th^ percentile. Whiskers represent the lowest values and the highest values of skin humidity. Filled circles represent outliers. AU: arbitrary unit. **d** LDA (linear discriminant analysis) plot of skin bacteria found by LEfSe showing their association with the host genotype at rs6996321. Cladogram on the right indicates the phylogenetic distribution of the skin bacteria. Each color represents host genotype: CC genotype in red and CT in green. Circles are arranged by phylogenetic levels from kingdom, phylum, class, family, genus, and species from inside out

We next explored associations between the skin microbiota composition and host genetic variation. For this investigation, we focused on SNPs in a panel of select host genes known to affect sebum production (*MC5R*, *FA2H*, *DGAT1*, *DGAT2*, *SCD1,* and *ELOVL4*), pigmentation (*DCT*, *OCA2*, and *TYRP1*), skin humidity (*AQP3*), skin barrier function (*KLF4, FLG, POF1B,* and *SPINK5*), and hair follicle development (*EDA1*, *EDAR*, *EDARADD*, and *PKP1*) (Additional file 1: Table S4). The selection of genes was performed manually. One of 275 SNPs was significantly associated with the abundance of a specific bacterial species (Table 3 and Fig. 4a), where significance was defined using a cut-off level of *P* < 0.05/275 = 0.000182. The identified SNP was localized in a gene known to play a role in skin barrier function (*FLG*). Marker rs6996321 (allele T) in *FLG* was negatively associated with *C. jeikeium,* although the abundance difference was small between the host genotypes (*P* < 0.05; Fig. 4b). *FLG* defects are known to cause allergic skin diseases, such as atopic eczema and ichthyosis vulgaris [32], both of which are characterized by dry skin. Correspondingly, possession of the minor allele T tended lower skin humidity but it was not statistically proved (Fig. 4c, *P* > 0.05). *FLG*, which encodes filaggrin, is a structural protein in the cornified envelop of the stratum corneum critical for skin barrier function [33]. Mutations in the serine protease St14 in mice, which impairs filaggrin processing, also results in an ichthyotic phenotype [34]. Importantly, *Corynebacterium* is overrepresented in the skin microbiota of St14-deficient mice, suggestive of a possible link between bacteria and filaggrin processing and pathogenesis. The allelic association with bacterial abundance was further confirmed using LEfSe analysis, an algorithm based on calculation of significantly different phenotypes using the Kruskal-Willis test followed by the Wilcoxon rank-sum test for *post hoc* analysis (Fig. 4d) [35]. Linear Discriminant Analysis (LDA) scores allowed us to estimate the effect size of the differentially abundant features. LEfSe analysis showed an additional association between *Peptoniphilus asaccharolyticus* and the minor allele of *FLG*. Although not identified in the SNP analysis, a substantial percentage of *Peptoniphilus* abundance could be attributed to heritability, indicating a possible genetic relationship between the bacteria and host. Interactions between the host and the skin microbiota have been analyzed in association with multiple skin diseases [36–38]. For example, a recent quantitative trait locus (QTL) mapping of mouse skin microbiotas revealed a strong connection between disease susceptibility and the host genotype-dependent microbial composition [38]. This study, performed on a genome-wide scale, detected QTL regions related to innate immune activation. This and our investigation did not identify overlapping QTL regions, which is conceivable because our SNP analysis was based on the specifically selected gene categories with known implications for human skin structure and function. Beyond the identification of cis-regulatory element associated SNPs, recent SNP association studies are expanding to include flanking regions of the target genes and long-range enhancer/promoter [39–41]. In this way, it is able to discover the interplay between target SNPs and their transcriptional regulators and ultimately, practical roles of the SNPs. Further studies on such long-range SNPs should allow us deeper insights into the genetic associations on the skin microbiome.

**Table. 3:** Associations between the skin microbiota and SNPs of targeted human genes after adjustment for age and gender

Bacteria	Marker	Associated gene	Chromosome	Chromosome position	Minor allele	MAF^a^	β^b^	95 % CI	*P*-value	FDR_BH	LDA effect size^c^
Skin barrier function											
*Corynebacterium jeikeium*	rs6996321	*FLG*	8	38441503	T	0.4545	−0.5174	−0.8929, −0.1418	0.00017	0.03383	3.37707

The direction of the β value in Table 2 indicates the positive or negative association between the minor allele and the bacteria

^a^Minor allele frequency

^b^Regression coefficient

^c^Linear discriminant analysis effect size assessed using the LEfSe (LDA coupled with effect size measurements) software

## Conclusions

In this study, we describe host genetic factors and other host-intrinsic characteristics that directly influence the composition of the skin microbiota. We further demonstrated a strong association between the composition of skin microbiota and human genetic factors related to skin barrier function. Analysis of genetically identical MZ twins and half-identical DZ twins can be used to identify and discriminate genetic and environmental effects on the microbiome [42]. Investigation of the twin pairs proved that the skin microbiome is shaped both by host genetics and their environmental factors. Additionally, analysis of human genetic traits associated with the skin microbiota was performed using a candidate-gene approach. For this investigation, we chose five representative traits that affect dermatologic conditions; namely, sebum production, pigmentation, skin humidity, skin barrier function, and hair follicle formation. To confirm the results of the association analysis, the bacterial abundance associated with host alleles was further analyzed using LEfSe. One SNP, located in a gene related to skin barrier function, was significantly associated with *C. jeikeium. C. jeikeium* abundance was lower in subjects containing the minor allele of *FLG*, which encodes filaggrin, a structural protein in the cornified envelop of the stratum corneum critical for skin barrier function.

Although we have explored intrinsic characteristics of the human skin microbiome in aspect of host genetics and environmental factors, our study has statistical limitations from a small sample size. In genetic association studies, a sufficient number of samples are critical to detect causality between genes and phenotypes. Furthermore, the collective size of bacteria identified by microbiome analysis creates more stringent p-values to achieve asking for increased sample size to identify additional links between host genetic factors and the composition of the skin microbiota. To overcome such limitations, we tried to assess the genetic impacts put on the skin microbiome using different analytical approaches and the results are supportive of our findings. Yet, more expansive genome-wide association studies with increased sample size are warranted to identify additional links between host genetic factors and the composition of the skin microbiota. Our results provide insight into skin traits associated with individual’s microbiome and potential genomic and microbial targets for skin healthcare.

## Methods

### Study population and sample collection

Study subjects were either MZ or DZ twins, and their mothers were recruited for the Healthy Twin Study as part of the Korean Genome Epidemiology Study [43] between September 2010 and August 2011. Zygosity of twins was confirmed using either 16 short tandem repeat (STR) markers (15 autosomal markers and 1 sex-determining marker) (67 %) or a self-administered zygosity questionnaire (33 %), which showed >90 % accuracy [44]. A total of 51 trios were initially selected, but 6 subjects were excluded due to intake of antibiotics within 3 months of sample collection. Thus, the final sample size was 45 individuals including 8 MZ twin pairs, 4 DZ twin pairs, and 21 family members; the members were composed of 5 parent-offspring pairs and 11 mothers of the twin pairs. Age of the mothers ranged from 49–79 years, and twin children and singletons ranged from 26–55 years. Two subjects exhibited mild symptoms of atopy, but were not excluded as they were only reported to have such symptoms and not prescribed any medication. Dermatological phenotypes such as pigmentation and humidity were available from 32 participants. The participants provided their written informed consent to participate in this study. All experiments involving human subjects were approved by the Korea Centers for Disease Control and the Institutional Review Board of the Seoul National University (IRB No. 144–2011–07–11).

The inner wrist of the right arm was swabbed with two sterile cotton swabs moistened with ST solution (0.15 M NaCl with 0.1 % Tween 20) [45]. The number of strokes was 4–5 times per sampling with gentle pressure. The heads of the cotton swabs were stored in 100 μl of ST solution at −80 °C until use. Swab samples were collected after 3-hr medical checkup securing the time from contact with water and soap. Additionally, skin phenotype—including pigmentation and humidity—were determined using different probes with a C + K multi probe adapter MPA 9 (Courage + Khazaka Electronic GmbH, Köln, Germany) on the same date. Sample collection and visiting was done at set times strictly. Skin pigmentation was measured from the flexor surface of right arm using a Mexameter® MX 18, as described previously [46]. The device quantifies the ratio of light emitted and reflected by skin chromophores and calculates the amount of melanin. Melanin levels were expressed as the melanin index, which ranged between 0 and 150 arbitrary units (AU) for light skin tone, and 150 and 250 AU for mid-tone, skin pigmentation. For skin humidity, capacitance measurement was performed using a Corneometer®CM825 [47]. Each phenotype was measured three times at 18–23 °C and 40–60 % relative humidity. Corneometry values of greater than 45 AU indicate sufficiently moisturized skin, and values less than 45 AU were considered dry skin. The categories of each trait followed the manufacturer’s instructions. The average value of the repeated measurements was used unless the measurements differed by more than 10 %. In this case, the median of the three measurements was taken.

### DNA extraction and 454 pyrosequencing

Total genomic DNA was extracted following the bead-beating extraction protocol [48]. Briefly, the cotton swab and ST solution were added to 500 μL of extraction buffer (200 mM NaCl, 200 mM Tris, and 20 mM EDTA; pH 8), 500 μL of phenol:chloroform:isoamyl-alcohol (25:24:1; pH 7.9) (Sigma, Steinheim, Germany), 210 μL of 20 % SDS, and 500 μL of zirconia-silica beads (0.1 mm in diameter; Biospec Products Inc., Bartlesville, OK). The mixture was homogenized using a Vortex Adaptor (MoBio Laboratories, Solana Beach, CA) for 2 min at room temperature. DNA extraction was performed with 500 μL of phenol: chloroform: isoamyl-alcohol (25:24:1; pH 7.9), followed by isopropanol precipitation. The nucleic acid solutions were stored at −70 °C until use. The V2 and V3 regions of the 16S rRNA genes were amplified from total DNA, extracted, and pyrosequenced as described previously [49]. The experiments were quality controlled using proper negative and positive controls at the stage of DNA extraction and PCR amplification, respectively. The cotton swabs without swabbing the inner arm were used as the negative controls. PCR amplification of these controls did not amplify any bacterial DNA, thus they were not used for subsequent sequence analysis. The positive controls used were DNA from *E.coli* at the stage of PCR amplification. The sequence data have been submitted to the EMBL databases under accession number PRJEB5864 (http://www.ebi.ac.uk/ena).

### Bioinformatic analysis using 16S rRNA sequences

Sequence data were analyzed using the QIIME software package (version 1.5.0) [50]. Before the sequence analysis, quality filtering was performed including removal of low-quality sequences (<200 bp) and ambiguous reads and end-trimming. Subsequently, homopolymers were removed by denoising the sequence data set in the QIIME pipeline [51]. Representative sequence sets were chosen using UCLUST and clustered at the 97 % sequence similarity level. Processed sequences were aligned using PyNAST [52], and taxonomy was assigned using the ribosomal database project (RDP) classifier [53], and the Greengenes Database (gg_97_otus_4feb2011.fasta) was used as the reference [54]. The minimum confidence score for the taxonomy assignment to sequences was 0.8. Chimera sequences (10.55 %) were excluded from downstream analyses prior to the generation of phylogenic trees or OTU tables using ChimeraSlayer algorithm [55]. Bacterial diversity both within and between samples was assessed through alpha using Chao1 measure [56] and beta diversity using weighted and unweighted UniFrac distances [57]. Phylogenetic tree was generated using the FastTree method [58]. UniFrac metrics were also used to identify differences between sample pairs. Analysis of similarity (ANOSIM) was performed to test the statistical significance in the differences. Functional traits were determined from 16S-rRNA-based sequences using PICRUSt-0.9.1(http://picrust.github.io/picrust/) [14]. Unclassified pathways were excluded from the functional analysis.

### Heritability analysis

Heritability estimates of each skin microbe were calculated by variance component methods using Sequential Oligogenic Linkage Analysis Routines (SOLAR, version 6.6.2; Southwest Foundation for Biomedical Research, San Antonio, TX, USA) [59]. Skin bacterial abundances at all taxonomic levels were used as quantitative traits. As the bacterial abundances were not normally distributed, inverse normal transformation was applied to the traits before the heritability analysis. Additionally, the monozygotic twins were separately categorized from the rest of participants. SOLAR uses a maximum-likelihood method, which allows incorporation of fixed covariate effects (age, gender, and interaction between age and gender). Also, the software allows working with extended families and complicated pedigree of different age and sex. Variance component analysis decomposes the total variation (σp^2^) into additive genetic effects (σa^2^) and residual non-genetic variance (individual effect; σe^2^), which can be further specified to unmeasured common environmental effects (σc^2^). We fitted the variance component model into additive genetic and non-genetic components and added the common environmental variance when the effects (σc^2^) were present (σp^2^ = σa^2^ + σc^2^ + σe^2^). Thus, the heritability was estimated as the ratio of variance attributed to the additive genetic components and the total variance (σa^2^ /σp^2^).

### Skin traits associated with SNPs

For SNP genotyping, venous blood from each subject was collected and genomic DNA was extracted using the i-genomic Clinic DNA Extraction Kit (Intron, Seongnam, South Korea). Genotyping was performed on Affymetrix Genome Wide Human SNP Array 6.0 **(**Affymetrix, Inc., Santa Clara, CA). Exclusion criteria included SNPs with Minor Allele Frequency < 0.01, Hardy Weinberg Equilibrium < 0.001, low call rate (<95 %), Mendelian error, and non-Mendelian error. Mendelian and non-Mendelian errors were identified using the PEDSTATS 0.6.12 [60] and Merlin 1.1.2 [61] software.

Association analysis of skin microbiotas was performed using Plink 1.07 (http://pngu.mgh.harvard.edu/purcell/plink/) [62], as described previously [63]. Briefly, the QFAM (family-based test of association for quantitative traits) model was used to analyze SNPs obtained from skin-trait related genes (Additional file 1: Table S4). Candidate genes were selected based on their roles in regulatory functions or skin metabolism. The list of SNPs was obtained from the Single Nucleotide Polymorphism Database (dbSNP; Build 137) [64]. Skin bacterial abundances at all taxonomic levels were used as quantitative traits. QFAM uses a simple linear regression and corrects for family structure based on adaptive permutation; in this study, 100,000 permutations were performed. This adaptive permutation also alleviates the assumption about normality of data set. Prior to analysis, the bacterial abundances were adjusted for age and gender by fitting to a regression model in R version 3.0.2 [65], as QFAM does not allow for covariates. For MZ twins, only one individual from each pair was analyzed. The alleles of the significant SNPs were examined with the bacterial abundances using LEfSe (linear discriminant analysis [LDA] coupled with effect size measurements) software [35]. The linear discriminant analysis (LDA) effect size greater than 2 was used as the threshold for discriminative bacteria.

### Statistical analysis

Species richness and UniFrac distances were analyzed by the Wilcoxon rank sum test (two-tailed) using R version 3.0.2 [65]. Correlation test of skin pigmentation was performed by the Pearson correlation with the SPSS software, ver. 21 (Armonk, NY, US). Association of host genotype with skin humidity was tested using the Kruskal-Willis test.

## Additional file

Additional file 1: Figure S1. Number of OTUs by skin pigmentation. Boxes represent the 25^th^ percentile, median, and 75^th^ percentile. Whiskers represent the lowest values and the highest values of skin pigmentation. Filled circles represent outliers. MI: Melanin Index. Figure S2. Differences in skin microbial diversity. Principal coordinate analysis (PCoA) for (A) age, (B) gender (C) skin humidity (CV, Corneomerty value), and (D) pigmentation (MI, Melanin Index) based on 16S rRNA genes (weighted/unweighted UniFrac distances). Left: weighted, Right: unweighted measures. Table S1. Functional traits and their abundance based on the 16S rRNA genes of members of the skin microbiota. Table S2. Nearest Sequenced Taxon Index (NSTI) values for individuals. Table S3. Correlation between skin pigmentation and selected variables. Table S4. Summary of the SNPs of 14 targeted genes (DOCX 871 kb).
