# Supplementary material for: Genetic associations and shared environmental effects on the skin microbiome of Korean twins
Source: BMC Genomics. 2015 Nov 23;16:992. doi: 10.1186/s12864-015-2131-y (PMC4657342; doi:10.1186/s12864-015-2131-y)
Supplement: Additional file 1: Figure S1. — Number of OTUs by skin pigmentation. Boxes represent the 25th percentile, median, and 75th percentile. Whiskers represent the lowest values and the highest values of skin pigmentation. Filled circles represent outliers. MI: Melanin Index. Figure S2. Differences in skin microbial diversity. Principal coordinate analysis (PCoA) for (A) age, (B) gender (C) skin humidity (CV, Corneomerty value), and (D) pigmentation (MI, Melanin Index) based on 16S rRNA genes (weighted/unweighted UniFrac distances). Left: weighted, Right: unweighted measures. Table S1. Functional traits and their abundance based on the 16S rRNA genes of members of the skin microbiota. Table S2. Nearest Sequenced Taxon Index (NSTI) values for individuals. Table S3. Correlation between skin pigmentation and selected variables. Table S4. Summary of the SNPs of 14 targeted genes (DOCX 871 kb). [file 12864_2015_2131_MOESM1_ESM.docx]

**Figure S1.** Number of OTUs by skin pigmentation. Boxes represent the 25^th^ percentile, median, and 75^th^ percentile. Whiskers represent the lowest values and the highest values of skin pigmentation. Filled circles represent outliers. MI: Melanin Index.

**Figure S2.** Differences in skin microbial diversity. Principal coordinate analysis (PCoA) for (A) age, (B) gender (C) skin humidity (CV, Corneomerty value), and (D) pigmentation (MI, Melanin Index) based on 16S rRNA genes (weighted/unweighted UniFrac distances). Left: weighted, Right: unweighted measures.


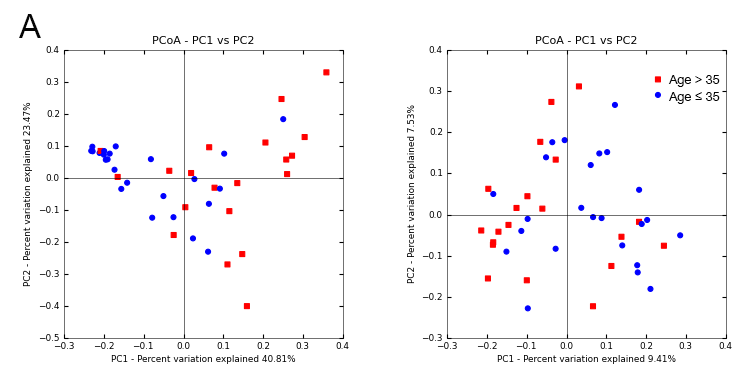


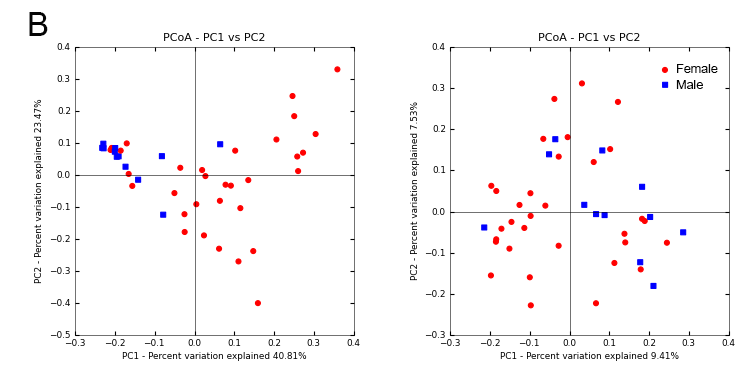


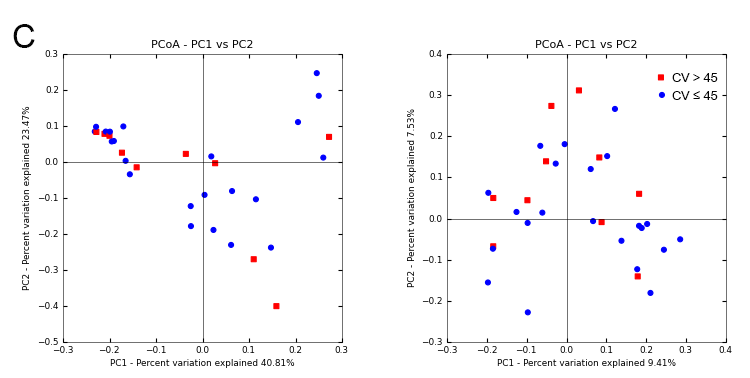


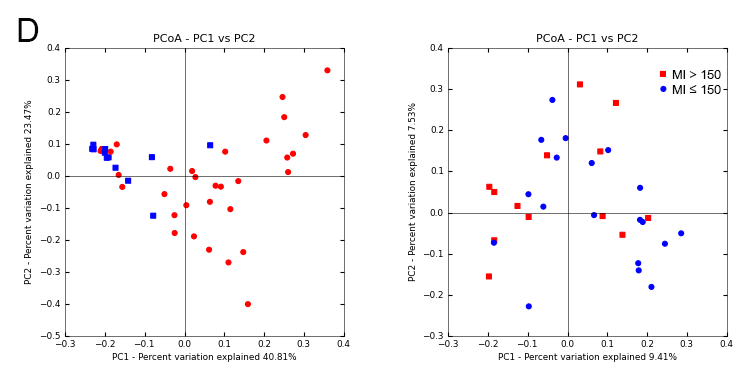


**Table S1.** Functional traits and their abundance based on the 16S rRNA genes of members of the skin microbiota.

| **Functional category** |  | **Functional trait abundance** |
| --- | --- | --- |
| Cell Growth and Death | Apoptosis | 16218 |
|  | Cell cycle - Caulobacter | 453537 |
|  | Meiosis - yeast | 87644 |
|  | p53 signaling pathway | 342 |
| Cell Motility | Bacterial chemotaxis | 549609 |
|  | Flagellar assembly | 330435 |
|  | Bacterial motility proteins | 81441 |
| Transport and Catabolism | Lysosome | 338521 |
|  | Peroxisome | 11246 |
| Membrane Transport | ABC transporters | 13553279 |
|  | Phosphotransferase system (PTS) | 2250192 |
|  | Secretion system | 4807466 |
|  | Transporters | 9998560 |
| Signal Transduction | MAPK signaling pathway - yeast | 65385 |
|  | Phosphatidylinositol signaling system | 207440 |
|  | Two-component system | 2315341 |
| Signaling Molecules and Interaction | Bacterial toxins | 769137 |
|  | CAM ligands | 34 |
|  | Cellular antigens | 38856 |
|  | G protein-coupled receptors | 24 |
|  | Ion channels | 165214 |
| Folding, Sorting and Degradation | Chaperones and folding catalysts | 3126409 |
|  | Proteasome | 26258 |
|  | Protein processing in endoplasmic reticulum | 46508 |
|  | RNA degradation | 402756 |
|  | Sulfur relay system | 736431 |
|  | Ubiquitin system | 45635 |
| Replication and Repair | Base excision repair | 1865982 |
|  | Chromosome | 3514619 |
|  | DNA repair and recombination proteins | 2255651 |
|  | DNA replication | 577441 |
|  | Homologous recombination | 1701352 |
|  | Mismatch repair | 1195936 |
|  | Non-homologous end-joining | 26241 |
|  | Nucleotide excision repair | 749408 |
| Transcription and Translation | Spliceosome | 4 |
|  | Transcription factors | 4583616 |
|  | Transcription machinery | 1307156 |
|  | Aminoacyl-tRNA biosynthesis | 4409418 |
|  | RNA transport | 167634 |
|  | Ribosome | 8942013 |
|  | Ribosome Biogenesis | 4424446 |
|  | Translation factors | 1544073 |
|  | mRNA surveillance pathway | 34 |
| Human Diseases | Bladder cancer | 97261 |
|  | Colorectal cancer | 41528 |
|  | Pathways in cancer | 140931 |
|  | Systemic lupus erythematosus | 1592 |
|  | African trypanosomiasis | 87 |
|  | Amoebiasis | 18556 |
|  | Bacterial invasion of epithelial cells | 465 |
|  | Epithelial cell signaling in Helicobacter pylori infection | 96 |
|  | Influenza A | 159160 |
|  | Measles | 62 |
|  | Pathogenic Escherichia coli infection | 38 |
|  | Pertussis | 1383 |
|  | Shigellosis | 48 |
|  | Staphylococcus aureus infection | 83292 |
|  | Vibrio cholerae infection | 12327 |
|  | Alzheimer's disease | 6474 |
|  | Amyotrophic lateral sclerosis (ALS) | 149678 |
|  | Huntington's disease | 169504 |
|  | Prion diseases | 32679 |
| Amino Acid Metabolism | Alanine, aspartate and glutamate metabolism | 1433539 |
|  | Amino acid related enzymes | 946198 |
|  | Arginine and proline metabolism | 1879213 |
|  | Cysteine and methionine metabolism | 2544361 |
|  | Glycine, serine and threonine metabolism | 1499951 |
|  | Histidine metabolism | 1673357 |
|  | Lysine metabolism | 2714906 |
|  | Phenylalanine metabolism | 2705497 |
|  | Tryptophan metabolism | 160490 |
|  | Tyrosine metabolism | 730080 |
|  | Valine, leucine and isoleucine biosynthesis | 486636 |
| Biosynthesis of Other Secondary Metabolites | Caffeine metabolism | 11729 |
|  | Clavulanic acid biosynthesis | 44 |
|  | Flavonoid biosynthesis | 14561 |
|  | Indole alkaloid biosynthesis | 1216 |
|  | Isoflavonoid biosynthesis | 47 |
|  | Isoquinoline alkaloid biosynthesis | 196561 |
|  | Penicillin and cephalosporin biosynthesis | 98304 |
|  | Phenylpropanoid biosynthesis | 73357 |
|  | Streptomycin biosynthesis | 170926 |
|  | Tropane, piperidine and pyridine alkaloid biosynthesis | 288206 |
|  | beta-Lactam resistance | 107290 |
| Carbohydrate Metabolism | Amino sugar and nucleotide sugar metabolism | 4754800 |
|  | Ascorbate and aldarate metabolism | 200443 |
|  | Butanoate metabolism | 949039 |
|  | C5-Branched dibasic acid metabolism | 1210453 |
|  | Citrate cycle (TCA cycle) | 2302433 |
|  | Fructose and mannose metabolism | 902393 |
|  | Galactose metabolism | 202030 |
|  | Glycolysis / Gluconeogenesis | 1643052 |
|  | Glyoxylate and dicarboxylate metabolism | 1145733 |
|  | Inositol phosphate metabolism | 768240 |
|  | Pentose and glucuronate interconversions | 4433086 |
|  | Propanoate metabolism | 2299451 |
|  | Pyruvate metabolism | 822297 |
|  | Starch and sucrose metabolism | 1003117 |
| Energy Metabolism | Carbon fixation in photosynthetic organisms | 225537 |
|  | Carbon fixation pathways in prokaryotes | 435426 |
|  | Methane metabolism | 281260 |
|  | Nitrogen metabolism | 520158 |
|  | Oxidative phosphorylation | 3637642 |
|  | Photosynthesis proteins | 1484734 |
|  | Sulfur metabolism | 255114 |
| Enzyme Families | Cytochrome P450 | 4876 |
|  | Peptidases | 4933229 |
|  | Protein kinases | 1408506 |
| Glycan Biosynthesis and Metabolism | Glycosaminoglycan degradation | 1879 |
|  | Glycosphingolipid biosynthesis - globo series | 22396 |
|  | Glycosylphosphatidylinositol(GPI)-anchor biosynthesis | 145 |
|  | Glycosyltransferases | 1086467 |
|  | Lipopolysaccharide biosynthesis proteins | 371995 |
|  | Other glycan degradation | 572941 |
|  | Peptidoglycan biosynthesis | 907124 |
|  | Various types of N-glycan biosynthesis | 240 |
| Lipid Metabolism | Arachidonic acid metabolism | 79707 |
|  | Ether lipid metabolism | 1054 |
|  | Fatty acid biosynthesis | 1436127 |
|  | Fatty acid elongation in mitochondria | 4 |
|  | Fatty acid metabolism | 440731 |
|  | Glycerolipid metabolism | 683818 |
|  | Glycerophospholipid metabolism | 1893576 |
|  | Linoleic acid metabolism | 223092 |
|  | Lipid biosynthesis proteins | 440297 |
|  | Primary bile acid biosynthesis | 53679 |
|  | Sphingolipid metabolism | 1087 |
|  | Steroid biosynthesis | 98368 |
|  | Synthesis and degradation of ketone bodies | 134470 |
| Metabolism of Cofactors and Vitamins | Biotin metabolism | 620838 |
|  | Folate biosynthesis | 1276185 |
|  | Lipoic acid metabolism | 331377 |
|  | Nicotinate and nicotinamide metabolism | 1502025 |
|  | One carbon pool by folate | 1689558 |
|  | Pantothenate and CoA biosynthesis | 1407009 |
|  | Porphyrin and chlorophyll metabolism | 4480856 |
|  | Retinol metabolism | 1049 |
|  | Riboflavin metabolism | 1214712 |
|  | Thiamine metabolism | 2043329 |
|  | Ubiquinone and other terpenoid-quinone biosynthesis | 863042 |
|  | Vitamin B6 metabolism | 401323 |
| Metabolism of Other Amino Acids | Cyanoamino acid metabolism | 703633 |
|  | D-Alanine metabolism | 538118 |
|  | D-Arginine and D-ornithine metabolism | 17332 |
|  | D-Glutamine and D-glutamate metabolism | 157365 |
|  | Glutathione metabolism | 259532 |
|  | Phosphonate and phosphinate metabolism | 202506 |
|  | Selenocompound metabolism | 15304 |
|  | Taurine and hypotaurine metabolism | 194955 |
|  | beta-Alanine metabolism | 161612 |
| Metabolism of Terpenoids and Polyketides | Biosynthesis of 12-, 14- and 16-membered macrolides | 2 |
|  | Biosynthesis of siderophore group nonribosomal peptides | 374811 |
|  | Biosynthesis of type II polyketide backbone | 8445 |
|  | Carotenoid biosynthesis | 82333 |
|  | Geraniol degradation | 481000 |
|  | Limonene and pinene degradation | 1242560 |
|  | Polyketide sugar unit biosynthesis | 349102 |
|  | Prenyltransferases | 1101233 |
|  | Sesquiterpenoid biosynthesis | 70 |
|  | Terpenoid backbone biosynthesis | 1142596 |
| Nucleotide Metabolism | Purine metabolism | 2436871 |
|  | Pyrimidine metabolism | 6069608 |
| Xenobiotics Biodegradation and Metabolism | 1,1,1-Trichloro-2,2-bis(4-chlorophenyl)ethane (DDT) degradation | 378 |
|  | Aminobenzoate degradation | 355000 |
|  | Atrazine degradation | 155446 |
|  | Benzoate degradation | 199806 |
|  | Bisphenol degradation | 147196 |
|  | Caprolactam degradation | 121723 |
|  | Chloroalkane and chloroalkene degradation | 72900 |
|  | Chlorocyclohexane and chlorobenzene degradation | 122381 |
|  | Dioxin degradation | 180149 |
|  | Drug metabolism - cytochrome P450 | 11015 |
|  | Drug metabolism - other enzymes | 562476 |
|  | Ethylbenzene degradation | 363317 |
|  | Fluorobenzoate degradation | 61633 |
|  | Metabolism of xenobiotics by cytochrome P450 | 209384 |
|  | Naphthalene degradation | 2852 |
|  | Nitrotoluene degradation | 110279 |
|  | Polycyclic aromatic hydrocarbon degradation | 72165 |
|  | Styrene degradation | 15480 |
|  | Toluene degradation | 849806 |
|  | Xylene degradation | 264765 |
| Organismal Systems | Cardiac muscle contraction | 51452 |
|  | Bile secretion | 10785 |
|  | Carbohydrate digestion and absorption | 80 |
|  | Mineral absorption | 29918 |
|  | Pancreatic secretion | 15 |
|  | Adipocytokine signaling pathway | 355476 |
|  | GnRH signaling pathway | 2114 |
|  | Insulin signaling pathway | 148848 |
|  | Melanogenesis | 228 |
|  | PPAR signaling pathway | 35533 |
|  | Circadian rhythm - plant | 15091 |
|  | Plant-pathogen interaction | 330990 |
|  | Proximal tubule bicarbonate reclamation | 71595 |
|  | Vasopressin-regulated water reabsorption | 18760 |
|  | NOD-like receptor signaling pathway | 2260 |
|  | Cholinergic synapse | 95 |
|  | Glutamatergic synapse | 304997 |

**Table S2.** Nearest Sequenced Taxon Index (NSTI) values for individuals.

| **Individual** | **NSTI score** |
| --- | --- |
| Mother 1 | 0.08 |
| MZ 1 (1) | 0.03 |
| MZ 1 (2) | 0.03 |
| Mother 2 | 0.06 |
| MZ 2 (1) | 0.03 |
| MZ 2 (2) | 0.03 |
| Mother 3 | 0.02 |
| MZ 3 (1) | 0.02 |
| MZ 3 (2) | 0.02 |
| Mother 4 | 0.07 |
| MZ 4 (1) | 0.03 |
| MZ 4 (2) | 0.03 |
| Mother 5 | 0.11 |
| MZ 5 (1) | 0.03 |
| MZ 5 (2) | 0.04 |
| Mother 6 | 0.07 |
| MZ 6 (1) | 0.02 |
| MZ 6 (2) | 0.02 |
| Mother 7 | 0.03 |
| MZ 7 (1) | 0.02 |
| MZ 7 (2) | 0.02 |
| Mother 8 | 0.05 |
| MZ 8 (1) | 0.09 |
| MZ 8 (2) | 0.03 |
| Mother 9 | 0.02 |
| MZ 9 | 0.02 |
| Mother 10 | 0.03 |
| MZ 10 | 0.07 |
| Mother 11 | 0.04 |
| MZ 11 | 0.02 |
| Mother 12 | 0.04 |
| DZ 12 (1) | 0.02 |
| DZ 12 (2) | 0.03 |
| Mother 13 | 0.04 |
| DZ 13 (1) | 0.04 |
| DZ 13 (2) | 0.06 |
| Mother 14 | 0.04 |
| DZ 14 (1) | 0.03 |
| DZ 14 (2) | 0.03 |
| Mother 15 | 0.05 |
| DZ 15 | 0.05 |
| Mother 16 | 0.21 |
| DZ 16 | 0.02 |
| DZ 17 (1) | 0.04 |
| DZ 17 (2) | 0.07 |

**Table S3.** Correlation between skin pigmentation and selected variables.

|  |  |  |
| --- | --- | --- |
|  | Pearson coefficient | P-value |
| Age | 0.234 | 0.205 |
| Sex | -0.031 | 0.868 |
| Sebum production | 0.196 | 0.290 |
| Skin humidity | 0.393 | 0.029 |

**Table S4.** Summary of the SNPs of 14 targeted genes.

| **Category** | **Gene** | **SNPs** |
| --- | --- | --- |
| Sebum | MC5R | rs1541276, rs11080686 |
|  | FA2H | rs7192551, rs2240244, rs6564162, rs6564163, rs2240252, rs11863417, rs4488477, rs11640870, rs6564166, rs8048578, rs8054687, rs8060979 |
|  | DGAT1 | rs3757973, rs3757971 |
|  | DGAT2 | rs10899118, rs10899121, rs11236531, rs12222995, rs1017713 |
|  | ELOVL4 | rs2991, rs7743696, rs9448857, rs865264, rs12196014, rs7754628, rs239516 |
|  | SCD1 | rs639060, rs612472, rs522951, rs3870747, rs3071, rs3793766, rs3793768, rs599961, rs560792 |
| Pigmentation | DCT | rs17791924, rs1325611, rs3782972, rs9516416, rs9516418, rs9524502, rs9524503 |
|  | OCA2 | rs16950402, rs7164752, rs8034368, rs17651026, rs1909266, rs3930739, rs17674023, rs12592159, rs4778121, rs4778185, rs17674249, rs10162623, rs8031191, rs8035334, rs4632082, rs12592307, rs6497248, rs8025804, rs1004611, rs768547, rs768546, rs4778199, rs4778200, rs7177529, rs8036059, rs7161804, rs16950490, rs2703970, rs2594895, rs2253509, rs2594899, rs2594900, rs11854269, rs2703983, rs2703926, rs13329497, rs1037206, rs3751651, rs2703961, rs2703960, rs7172981, rs1349105, rs1375170, rs1800414, rs7170989, rs12593141, rs7165923, rs3884517, rs1448488, rs12910433, rs3794609, rs4778221, rs730502, rs8029026, rs2077596, rs12438490, rs2305252, rs3794602, rs4778232, rs1448485, rs16950821, rs8024968, rs7170869, rs12442147, rs1597196, rs6497254, rs895828, rs7184011, rs7179419, rs916977 |
|  | TYRP1 | rs2075508, rs2762462, rs10960756 |
| Humidity | AQP3 | rs17253713 |
| Skin barrier function | KLF4 | rs2236599 |
|  | FLG | rs2288696, rs11777067, rs7012413, rs6996321 |
|  | POF1B | rs17278401, rs6653050, rs12014113, rs6623262, rs363754, rs12012683, rs363776, rs12557215, rs363765, rs2343538 |
|  | SPINK5 | rs3756688, rs1423007, rs2287774, rs2287773, rs9325064, rs986494, rs1423001, rs12108690, rs4529181, rs6580520, rs6580521, rs1862446, rs2303066, rs2303068, rs2303069, rs1422987, rs3777142, rs988885, rs17107763, rs3815738, rs1862439, rs3777138, rs9325073, rs11743440, rs17704908, rs1422993, rs2112767, rs12187820, rs12188819 |
| Follicle development | EDA1 | rs1327346, rs5936735, rs5936736, rs5980841, rs2804385, rs2214067, rs5936753, rs6525337, rs5980665, rs11094136, rs760041, rs5936512, rs989059, rs1202990, rs1011153, rs3920912, rs5936781, rs5936783, rs5980666, rs1202987, rs1202986, rs1202978, rs2382874, rs5980881, rs5936802, rs5936806, rs5936807, rs6624450, rs5936809, rs5936811, rs5936812, rs6625544, rs17216448, rs17311385, rs6625549, rs1327477, rs5936814, rs6625553, rs5936818, rs6625561, rs1590687, rs7063936, rs5980890, rs2274469, rs2296765 |
|  | EDAR | rs3749096, rs3749097, rs6542783, rs13430741, rs260633, rs11123717, rs10203613, rs749810, rs6732336, rs12476308, rs899259, rs6542787, rs260709, rs260711, rs260699, rs260700, rs260705, rs5021634, rs10779899, rs10496426, rs7594209, rs260694, rs11691107, rs12992554, rs260603, rs260674, rs17269487, rs13022998, rs11687181 |
|  | EDARADD | rs645507, rs630891, rs1272860, rs665504, rs654862, rs1759388, rs634626, rs604304, rs639027, rs1253630, rs648236, rs646040, rs4659672, rs12089515, rs10925131, rs17582358, rs1253621, rs7512191, rs11587318, rs585273 |
|  | PKP1 | rs2268147, rs832164, rs832169, rs832174, rs700469, rs2268157, rs832148, rs10920169, rs1722780, rs1794868, rs1628556, rs1779293, rs854505, rs16848325, rs16848334, rs17425876, rs12120834, rs12143423, rs1046962 |
